# Improving Nanopore sequencing-based core genome MLST for global infection control: a strategy for GC-rich pathogens like *Burkholderia pseudomallei*

**DOI:** 10.1128/jcm.01569-24

**Published:** 2025-02-06

**Authors:** Sarah Weigl, Johanna Dabernig-Heinz, Fabian Granitz, Michaela Lipp, Laura Ostermann, Dag Harmsen, Thanh Trung Trinh, Ivo Steinmetz, Gabriel E. Wagner, Sabine Lichtenegger

**Affiliations:** 1 Diagnostic and Research Institute of Hygiene, Microbiology and Environmental Medicine, Diagnostic and Research Center for Molecular Biomedicine, Medical University of Graz 670030 , Graz, Austria; 2 Department of Periodontology and Operative Dentistry, University Hospital Münster 39069 , Münster, Germany; 3 VNU Institute of Microbiology and Biotechnology, Vietnam National University 597407 , Hanoi, Vietnam; Maine Medical Center Department of Medicine, Portland, Maine, USA

**Keywords:** WGS-based bacterial typing, Nanopore sequencing, core genome MLST, DNA methylation, *Burkholderia pseudomallei*, melioidosis, long read sequencing

## Abstract

Genomic surveillance of pathogens is essential to trace infections and analyze resistance markers. Core genome multilocus sequence typing (cgMLST) facilitates genomic surveillance by simplified analysis and standardization. However, its application is limited by the poor cost-efficiency of short-read (SR) sequencing. Oxford Nanopore long-read sequencing (ONT-LR), which allows fast on-site analysis with comparatively low costs, could provide an alternative. Despite ONT-LR raw read accuracy improvement, evidence for methylation-based errors accumulates. PCR-based library preparation, suggested as a solution, presumably poses difficulties for GC-rich bacteria. We challenged ONT-LR-based cgMLST using the highly GC-rich pathogen *Burkholderia pseudomallei* to develop a clinically applicable workflow. Our *B. pseudomallei* cgMLST scheme was applied to ONT-LR data, and the results were validated against SR data. Native, rapid, and PCR-based library preparation was performed and combined with different basecalling models (SUP@bacterial-methylation, SUP@v4.2, SUP@v4.3, and SUP@v5.0) and polishing strategies (medaka_consensus, medaka_variant, r103_min_high_g360). To ensure reliability across genotypes, we included 14 sequence types and 27 genotypes. The recommended ONT-LR workflow at study initiation (SUP@v4.2, medaka_consensus) showed nearly 200 allele differences compared with the reference for specific strains. PCR-based library preparation resulted in missing targets and typing errors of up to 21 alleles. Native barcoding with SUP@v5.0 basecalling and r103_min_high_g360 polishing outperformed the PCR-based approach in all parameters reducing the error rate to a maximum of two allele differences. The optimized ONT-LR-based cgMLST workflow for *B. pseudomallei* integrates high resolution and ease of implementation with enhanced cost-efficiency for rapid diagnostics. The developed protocol might serve as a guideline for other GC-rich pathogens.

**IMPORTANCE:**

This study highlights a significant advancement in genomic surveillance of bacterial pathogens, specifically addressing the challenges posed by the GC-rich species *Burkholderia pseudomallei*. Core genome multilocus sequence typing (cgMLST) is widely used for bacterial typing as it combines high resolution with simple implementation and standardization. To improve cost efficiency and thus accessibility, we changed the sequencing approach from Illumina short-read (SR) to Oxford Nanopore long-read sequencing (ONT-LR). ONT-LR-based cgMLST showed a very high error rate compared with SR-based cgMLST, most likely due to methylation-associated errors. PCR-based library preparation, which is proposed to correct these errors, did not achieve the required accuracy. In contrast, native barcoding with advanced basecalling and polishing strategies massively reduces allelic differences. This optimized ONT-LR cgMLST workflow provides a transformative solution for cost-efficient, high-resolution typing of *B. pseudomallei*. Furthermore, this study can serve as a guide for similarly challenging bacteria.

## INTRODUCTION

Genomic pathogen surveillance is essential for outbreak management, monitoring resistance markers, and understanding disease dynamics (1, 2). Thereby, it plays a key role in managing and mitigating the impact of infectious diseases (3–6). Whole genome sequencing (WGS)-based methods provide ultimate resolution for analyzing phylogenetic relationships and known virulence and resistance markers. Single nucleotide polymorphism-based methods provide high resolution but face the bottleneck of demanding high computational power and profound bioinformatics skills. By relying on gene-by-gene comparison of thousands of core genes, core genome multilocus sequence typing (cgMLST) ensures high resolution, effectively addresses the aforementioned issues, and offers a standardized method simplifying data exchange (7–10). Together with the availability of open-source tools (e.g., pyMLST [11]), cgMLST facilitates global pathogen surveillance and control (7).

One of the last obstacles, needed to be addressed, is poor cost-efficiency for low sample numbers and high instrument prices for gold standard short read sequencing (SR) (12, 13). Applying Oxford Nanopore technology´s long read (ONT-LR) sequencing could elevate the accessibility of cgMLST due to low equipment fees and cost-efficiency even for small sample numbers (14–16). For *Burkholderia pseudomallei*, a bacterium with a genome size exceeding 7 MB, sequencing a single strain costs approximately $158 using Illumina and around $74 using ONT sequencing. The cost of an Illumina MiSeq system ranges from $52,000 to $210,000 with significant annual maintenance expenses. An Oxford Nanopore MinION device is available for $1,990, including consumables.

Additional benefits of ONT-LR sequencing are the generation of long reads facilitating the assembly of circular chromosomes and plasmids (17) and real-time data generation, allowing fast, on-site analysis (12). Despite these advantages, its application was restricted by an increased error rate compared with SR methods. Improvement in data acquisition and processing substantially increased the raw read accuracy, now reaching q20 (18). Based on these advancements, ONT-based cgMLST could represent a highly effective tool for cost-efficient, high-resolution pathogen surveillance. However, currently, we face the constraint of some species showing intolerably high typing error rates (12, 19). Given its potential to advance genomic pathogen surveillance, this observation calls for validation and improvement.

Although previously ONT-LR errors were mainly associated with homopolymer regions (20), the issue shifted toward methylation-related basecalling issues (13, 21). PCR-based library preparation is proposed to eliminate these errors (22). However, particularly, species with a complex and GC-rich genome are presumably challenging for PCR-based methods. Furthermore, this approach introduces the limitations of increased hands-on time, DNA amplification-induced errors, bias toward specific regions, and fragmented genomes. Based on current advancements, native barcoding (NBK) in combination with suitable data processing pipelines might compensate DNA methylation-induced errors. The aim of this study was to comprehensively validate PCR-based and native library preparation in combination with advanced data processing for a GC-rich species. We selected the melioidosis-causing pathogen *B. pseudomallei* as a model organism since it has one of the most complex bacterial genomes with a GC content of 68%. Furthermore, *B. pseudomallei* genomes are most likely drastically under-represented in the ONT training set due to restricted LR sequence data availability and its status as a highly neglected pathogen (23, 24). Furthermore, it is of particular importance to validate ONT-based cgMLST for melioidosis since it especially affects low- and middle-income countries, which could massively benefit from a cost-efficient, highly accurate typing method.

We successfully validated different ONT-LR protocols for cgMLST and resolved the described limitations. By including 27 genotypes, we prove applicability of our approach across different genotypes, which is particularly important since strain and sequence type (ST) specific typing errors were described (13, 19). Importantly, we include SUP@v5.0, which was to the best of our knowledge not applied in previous studies.

## MATERIALS AND METHODS

### DNA isolation

Thirty *B. pseudomallei* strains comprising 14 different STs were used for this study. *B. pseudomallei* strains were cultivated aerobically on Columbia agar containing 5% sheep blood (BD Biosciences) at 37°C for 24 h. TTSS-1 PCR was performed on all isolates for species identification. DNA was isolated using a single bacterial colony and the NucleoSpin microbial DNA kit (Macherey-Nagel) according to the instructions with slight modifications to improve quality (25). DNA concentration was determined using the Qubit BR assay kit on a Qubit four fluorometer (Thermo Fisher Scientific).

### Library preparation

Library preparation for ONT-sequencing was performed using one of the following kits (Oxford Nanopore) according to the manufacturer’s instructions: (i) Native Barcoding Kit 24 V14 (Q20+) (SQK-NBD114.24), (ii) Rapid Barcoding Kit 24 V14 (Q20+) (SQK-RBK114.24), and (iii) Rapid PCR Barcoding Kit 24 V14 (SQK-RPB114.24), which was additionally performed with modified conditions (Q5 high-fidelity DNA polymerase and Q5 GC-enhancer) (New England Biolabs).

### ONT-LR sequencing and basecalling

Libraries were sequenced on R10.4.1 flow cells (Oxford Nanopore) according to the manufacturer´s instructions. Data were basecalled and demultiplexed using: res_dna_r10.4.1_e8.2_400bps_sup@2023–09-22_bacterial-methylation, Dorado v0.4.0 (SUP@bacterial-methylation); dna_r10.4.1_e8.2_400bps_sup@4.2.0, Dorado v0.6.2 (SUP@v.4.2); dna_r10.4.1_e8.2_400bps_sup@4.3.0, Dorado v0.6.2 (SUP@v4.3); and dna_r10.4.1_e8.2_400bps_sup@5.0.0, Dorado v0.7.2 (SUP@v5.0).

### ONT-LR *de novo* assembly

Quality filtering was performed during basecalling to a quality score higher than 10. The *de novo* assembler, Flye (v 2.9.3), was used for assembly (26) with a mean assembly coverage of 303 (range: 43–462) for NBK, 126 (range: 78–203) for RBK, and 174 (range: 37–359) for the PCR-based library preparation kit. The N50 per assembly was 3,902,603 on average (range: 2,570,667–7,255,579) for NBK; 3,578,963 (range: 2,569,128–4,263,711) for RBK; and 50,936 (range: 17,876–121,875) for the PCR-based library preparation kit. Polishing was carried out with medaka (v 1.11.3/v 1.12 for SUP@v5.0) using the respective models (Table 1). Where noted, prior racon polishing was performed (v 1.5.0/Minimap v2.26). A bash script with instructions on how to apply Flye assembly and Medaka polishing to ONT reads is available at the GitHub page “https://github.com/swmedhyg/flye-medaka_shell-script”.

**TABLE 1:** Applied medaka models^*a*^

Basecalling model	medaka_consensus	medaka_variant	medaka_g360_HAC
SUP@bacterial-methylation	r1041_e82_400bps_sup_v4.2.0	r1041_e82_400bps_sup_variant_v4.2.0	r103_min_high_g360
SUP@v4.2	r1041_e82_400bps_sup_v4.2.0	r1041_e82_400bps_sup_variant_v4.2.0	r103_min_high_g360
SUP@v4.3	r1041_e82_400bps_sup_v4.3.0	r1041_e82_400bps_sup_variant_v4.3.0	r103_min_high_g360
SUP@v5.0	r1041_e82_400bps_sup_v5.0.0	r1041_e82_400bps_sup_variant_v5.0.0	r103_min_high_g360

^*a*^ Each basecalling model was combined with medaka_consensus, variant and r103_min_high_g360 (medaka_g360_HAC).

### SR sequencing and assembly

Libraries were prepared using the Nextera XT library preparation kit (Illumina, Inc.) according to the manufacturer ´s recommendations. SR sequencing was performed on an Illumina MiSeq instrument using MiSeq reagent kit version 2 (Illumina, Inc) for a 2- by 250-bp paired-end sequencing run. Raw data were assembled with SKESA (27) (SeqSphere+, Ridom GmbH) (v 9.0.7) (28) using default parameters.

### cgMLST-based bacterial typing

cgMLST analysis was performed in Ridom SeqSphere+ (v 9.0.7) using our published *B. pseudomallei* cgMLST scheme (7). An integrated contamination check was performed for all isolates (Mash screen). Distance matrices were generated in SeqSphere+ by cross-comparison of individual alleles of found target genes. Minimum spanning trees were created in SeqSphere+ using default parameters. The option “pairwise ignore missing values” was selected for all analyses.

### cgSNP analysis

cgSNP analysis was performed in Ridom SeqSphere+ (v.9.0.7) using 3,879 core genome target genes of our published *B. pseudomallei* cgMLST scheme (7). An integrated contamination check was performed for all isolates (Mash screen). Minimum spanning trees were created in SeqSphere+ using default parameters.

### Statistics and data visualization

Figures were created using SeqSphere+ (Ridom GmbH) and RStudio (RStudio, Inc., v2023.12.1 + 402) in R (v3.6.3). GC content was analyzed with FastQC (v0.12.1).

## RESULTS

**Fig 1:**
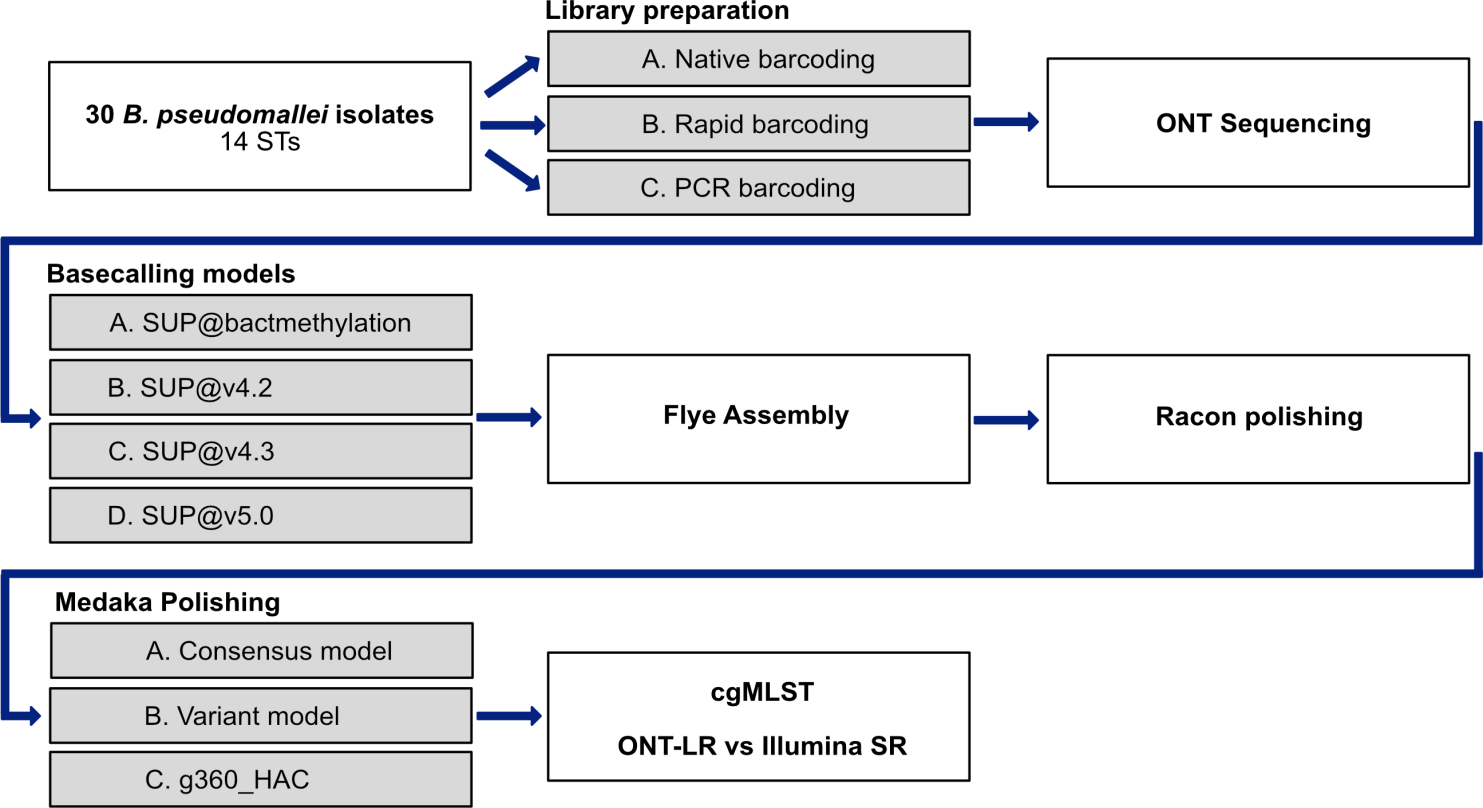
Flowchart of the applied ONT sequencing approaches and data processing pipelines. Gray boxes mark different alternatives. As readout, ONT-cgMLST was compared with SR-cgMLST.

To determine the optimal ONT-cgMLST pipeline for the GC-rich pathogen *B. pseudomallei*, we applied different library preparation protocols, basecalling models, and polishing strategies (Fig. 1). Furthermore, in a preliminary study, Ridom evaluated all available medaka polishing models with seven culture collection strains with finished NCBI RefSeq genomes (spanning the whole GC range). Those isolates were re-sequenced with ONT rapid barcoding libraries and basecalled with dorado. The g360_HAC medaka model outperformed all other models, which was therefore also included.

### Native barcoding using default parameters

**Fig 2:**
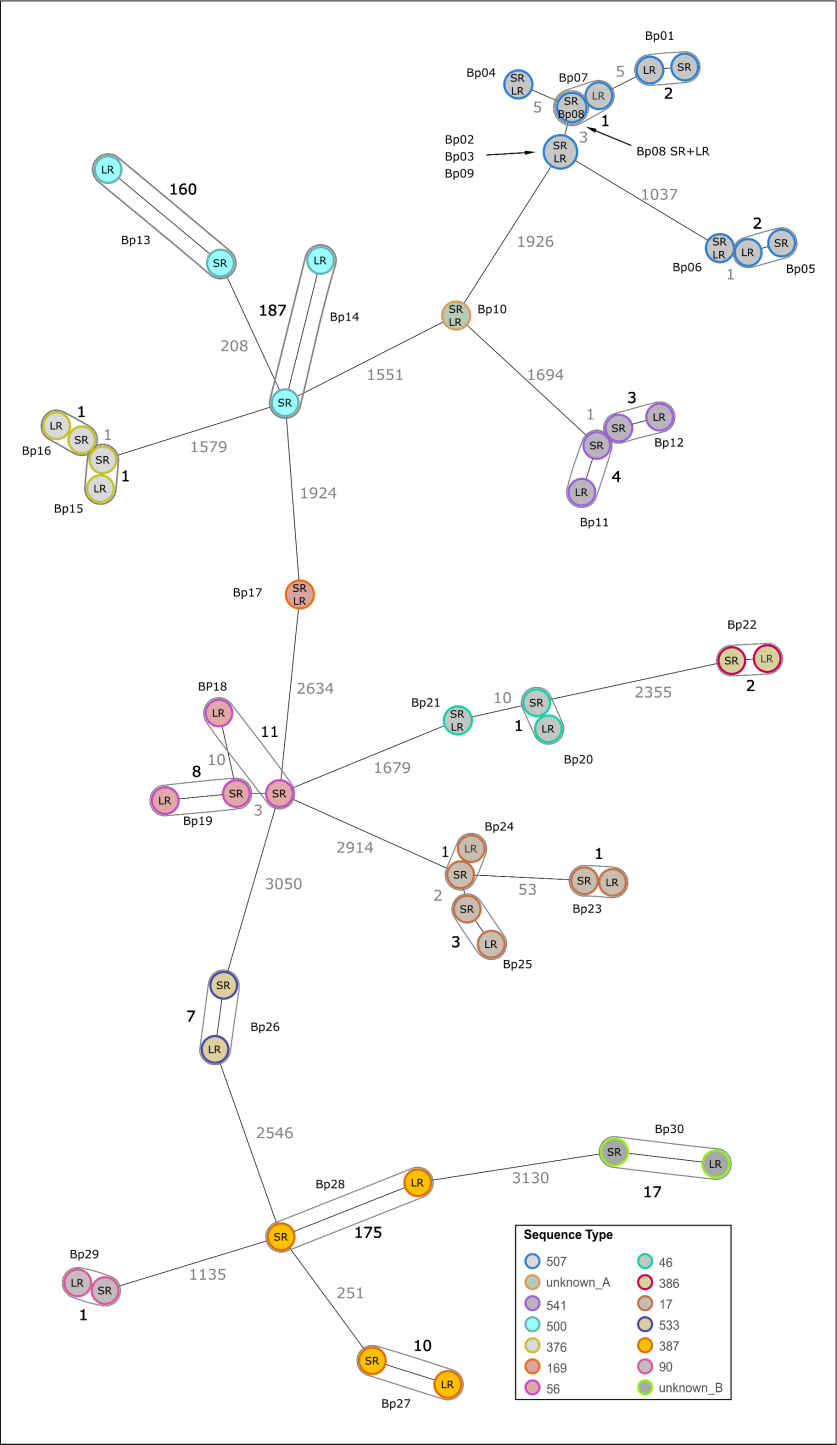
Comparison of ONT-LR cgMLST to the SR reference cgMLST. 30 *B. pseudomallei* strains (Bp01-Bp30) were analyzed using ONT-LR (SUP@4.2, medaka_consensus) and Illumina-SR based cgMLST. Each circle represents an allelic profile based on the allele calling of 4,221 target genes. The numbers on the connecting lines refer to the number of allele differences. Allele differences between different strains are shown in gray, whereas allele differences between SR and LR data of one particular isolate are shown in black. Colors represent different STs. When ONT-LR cgMLST does not match the Illumina-SR reference, nodes are circled. Bp, *B. pseudomallei*; LR, ONT-LR cgMLST; SR, Illumina SR cgMLST.

Prior to this study, no data on ONT-based *B. pseudomallei* cgMLST were available. Therefore, we initially validated NBK with the, at the commencement of this study, recommended workflow (SUP@4.2 with medaka_consensus). To account for genetic diversity and strain-specific methylation-related basecalling issues, we analyzed a diverse strain collection comprising 14 STs and 27 genotypes. For some strains, the ONT-cgMLST profile matched the SR-cgMLST reference (22/30: 0–4 differences; “low typing error strains”), whereas for others, we observed a medium (5/30: 7–17 differences; “medium typing error strains”) or high number of allele differences (3/30: 160–187 differences “high typing error strains”) (Fig. 2; Table S1). These results demonstrate that the implemented approach does not meet the criteria for high resolution typing. The observed discrepancy is strain-specific since strains from one certain ST display high typing errors (Bp13, and Bp14, ST500), whereas strains from another ST (Bp23, and Bp25, ST17) display low typing errors (Fig. 2; Table S1). This ST- and strain-specific discrepancy is in agreement with other emerging studies, where this observation was attributed to DNA methylation-associated basecalling issues (13, 22).

### PCR-based library preparation

**Fig 3:**
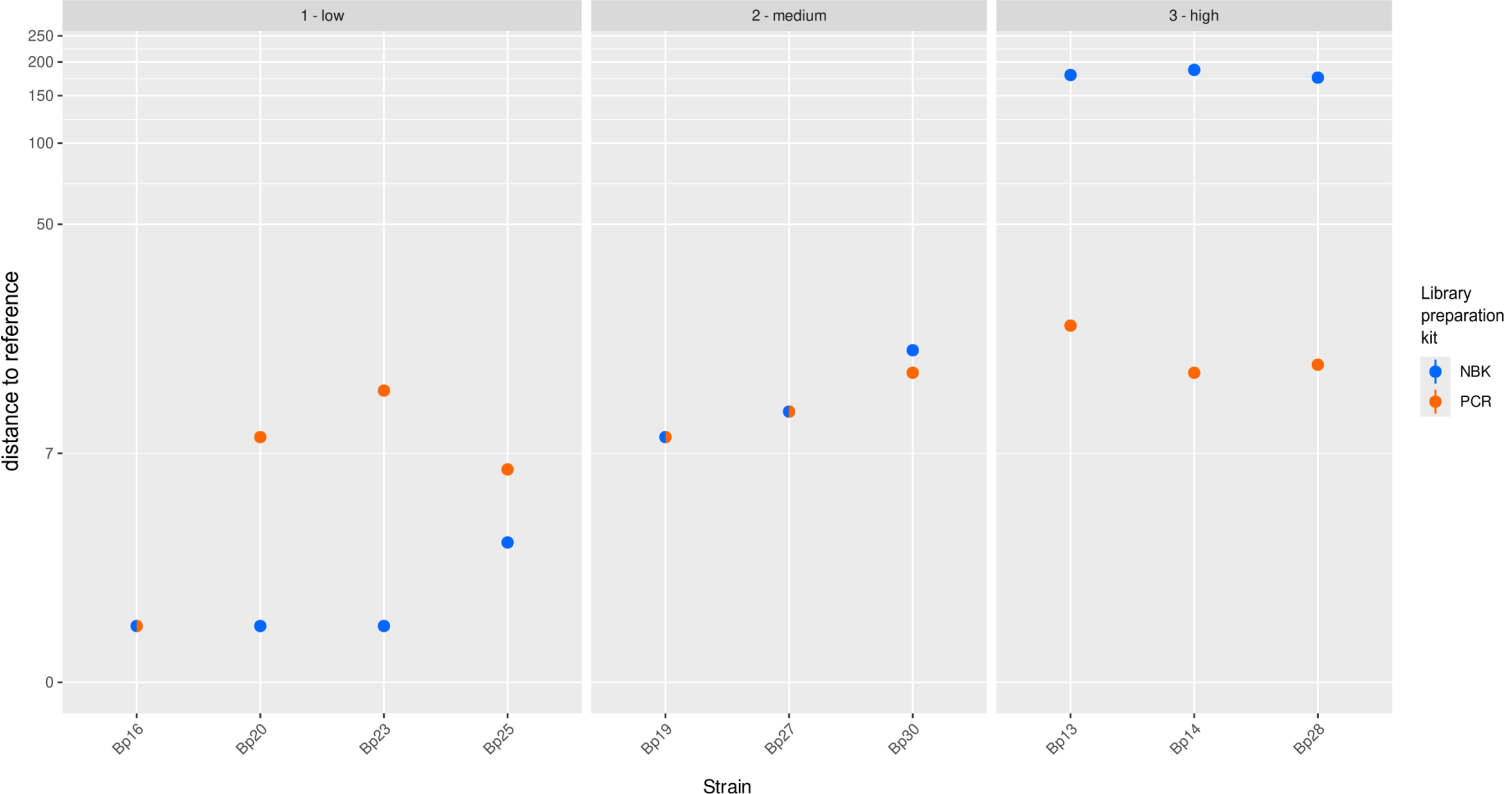
Allelic differences of the two ONT-cgMLST approaches (NBK-based and PCR-based), in comparison to the SR reference of the respective strain. Dots in blue show the allele differences of the NBK (SUP@4.2 with medaka_consensus) based ONT-cgMLST compared with the SR reference. Dots in orange show the allele difference of the PCR-based ONT-cgMLST compared with the SR reference. Mixed dots display overlapping results for the two ONT approaches. Bp, *B. pseudomallei*; NBK, native barcoding kit; PCR, PCR-based library preparation kit; low, “low typing error strains"; medium, “medium typing error strains”; high, “high typing error strains.”

To resolve this issue, PCR-based library preparation is suggested. The recommended protocol for PCR-based library preparation did not result in DNA amplification since no increase in amount was detected after PCR. Increasing the number of PCR cycles increased the DNA concentration but resulted in failed assemblies. We analyzed the generated reads and observed that the median GC content did not match the one of *B. pseudomallei* (Table S2), indicating a PCR bias toward AT-rich regions. We further adapted the protocol including a switch from Taq- to Q5-polymerase, the addition of GC enhancer, and Q5-optimized PCR conditions. We applied the adapted protocol to four “low typing error” (Bp20, Bp23, Bp16, and Bp25), three “medium typing error” (Bp30, Bp19 and and Bp27), and three “high typing error” (Bp13, Bp14, and Bp28) strains. This approach increased the GC-content from 58% to 64%−66%, almost reaching the one of native *B. pseudomallei* DNA (68%). As a result, this led to successful assemblies for all isolates. ONT-cgMLST analysis based on these data revealed a reduction in typing errors compared with NBK with default parameters. In detail, this approach resulted in a maximum of 21 allele differences compared with the SR reference (Fig. 3; Table S3). However, it increased the number of allele differences for previously unproblematic strains (e.g., for Bp23 from 1 to 12). Furthermore, it led to an increased number of contigs (average of 245.5) compared with the NBK (average of 2.5 contigs) and an increase in missing targets from 13 to 950 (average). This is above the 10% missing targets typically applied as threshold in cgMLST analysis.

### Native barcoding using different basecalling models

**Fig 4:**
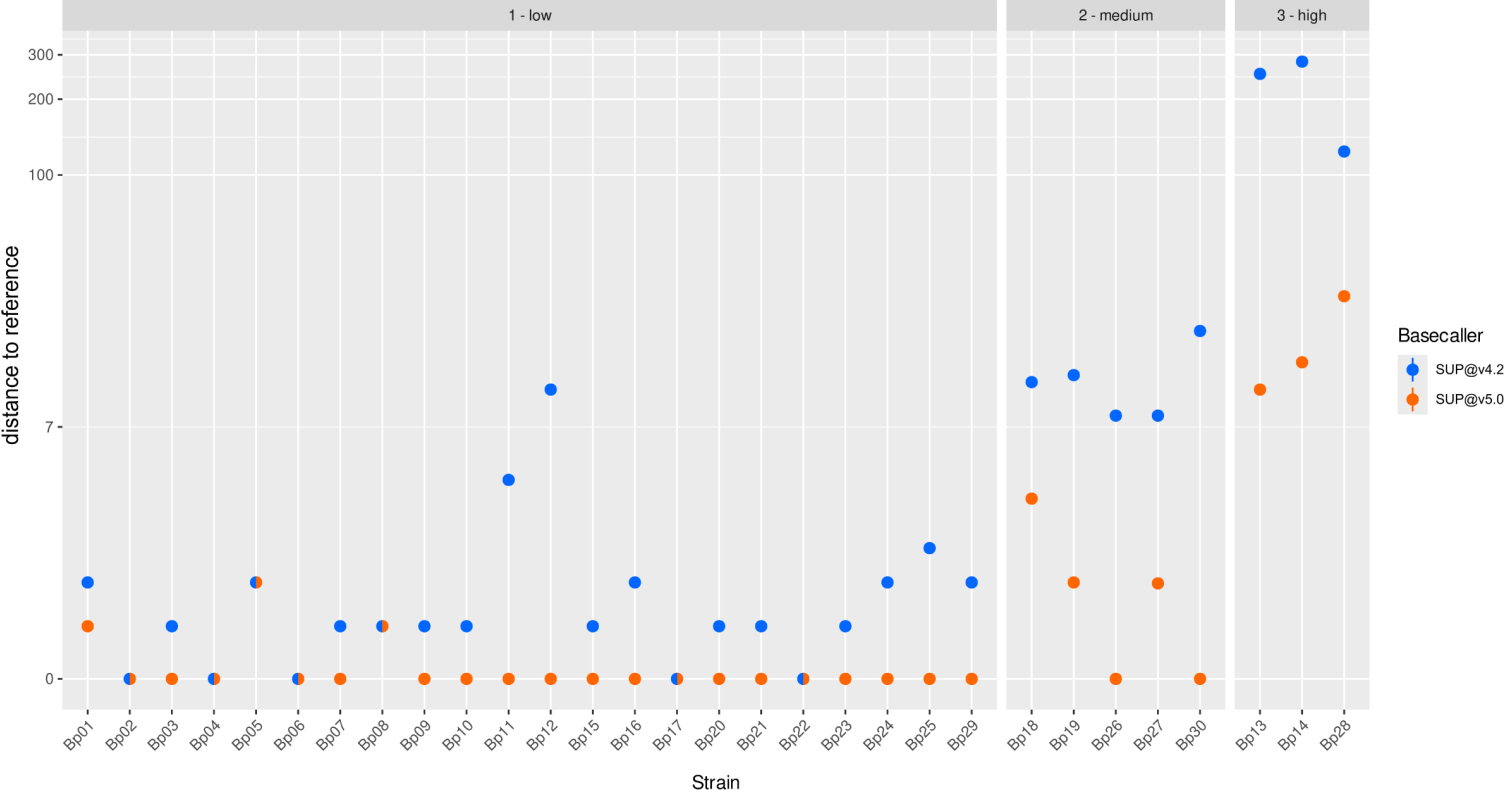
Allelic differences of the two NBK-based ONT-cgMLST methods in comparison to the SR reference. Dots in blue show the allele differences of the NBK (SUP@4.2)-based ONT-cgMLST compared with the SR reference. Dots in orange show the allele difference of the NBK (SUP@5.0)-based ONT-cgMLST compared with the SR reference. Mixed dots display overlapping results for the two ONT approaches. Bp, *B. pseudomallei*; NBK, native barcoding kit; PCR, PCR library preparation kit; low, “low typing error strains"; medium, “medium typing error strains”; high, “high typing error strains.”

Recently, new basecalling models became available, which could represent an alternative for reducing methylation-associated typing errors. Applying different basecalling models to all 30 data sets massively decreased the unpolished typing error rate compared with the initially implemented model (SUP@v4.2; Fig. 4; Table S4). SUP@bacterial-methylation resulted in 7.7, SUP@v4.3 in 3.9, and SUP@v5.0 in 2.6 mean allele differences compared with the SR reference. In contrast to the PCR-based library preparation protocol, there was no increase in allele differences for initially low typing error strains. Additionally, we observed a decrease in missing targets from an average of 18.1 for SUP@v4.2 (range: 1–138) to 8.4 for SUP@v5.0. (range: 1–48). In summary, applying novel basecalling models improved cgMLST-typing, with SUP@v5.0 providing the best results. Thereby, we were able to decrease errors for high typing error strains from originally 124 to 181 to a maximum of 33 allele differences.

### Native barcoding using different polishing strategies

**Fig 5:**
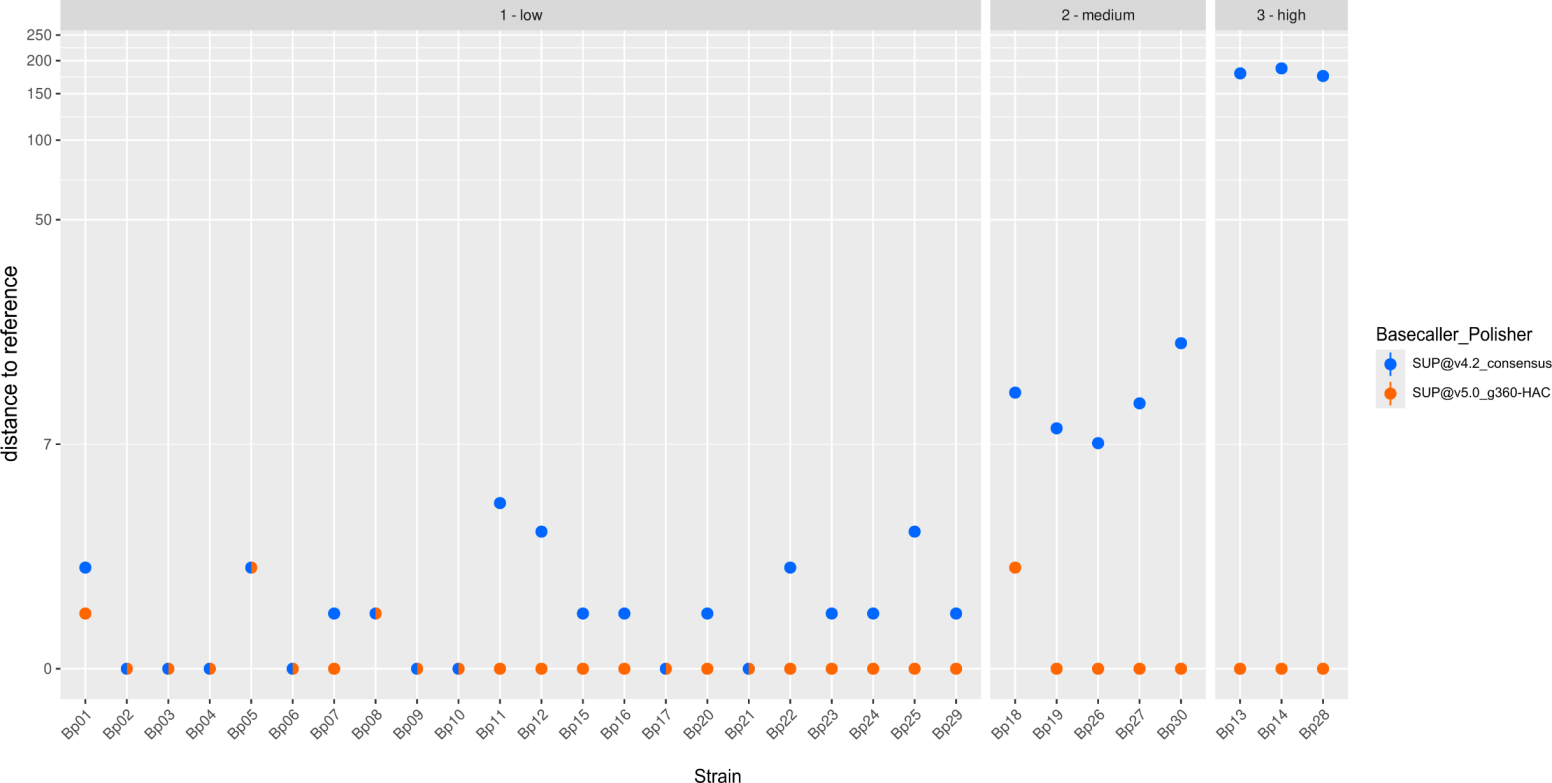
Allelic differences between the two NBK-based ONT-cgMLST methods in comparison to the SR reference. Dots in blue show the allele differences of the NBK (SUP@4.2 with medaka_consensus)-based ONT-cgMLST compared with the SR reference. Dots in orange show the allele difference of the NBK (SUP@5.0 with medaka_g360_HAC)-based ONT-cgMLST compared with the SR reference. Mixed dots display overlapping results for the two ONT approaches. Bp, *B. pseudomallei*; NBK, native barcoding kit; PCR, PCR library preparation kit; low, “low typing error strains"; medium, “medium typing error strains”; high, “high typing error strains.”

**Fig 6:**
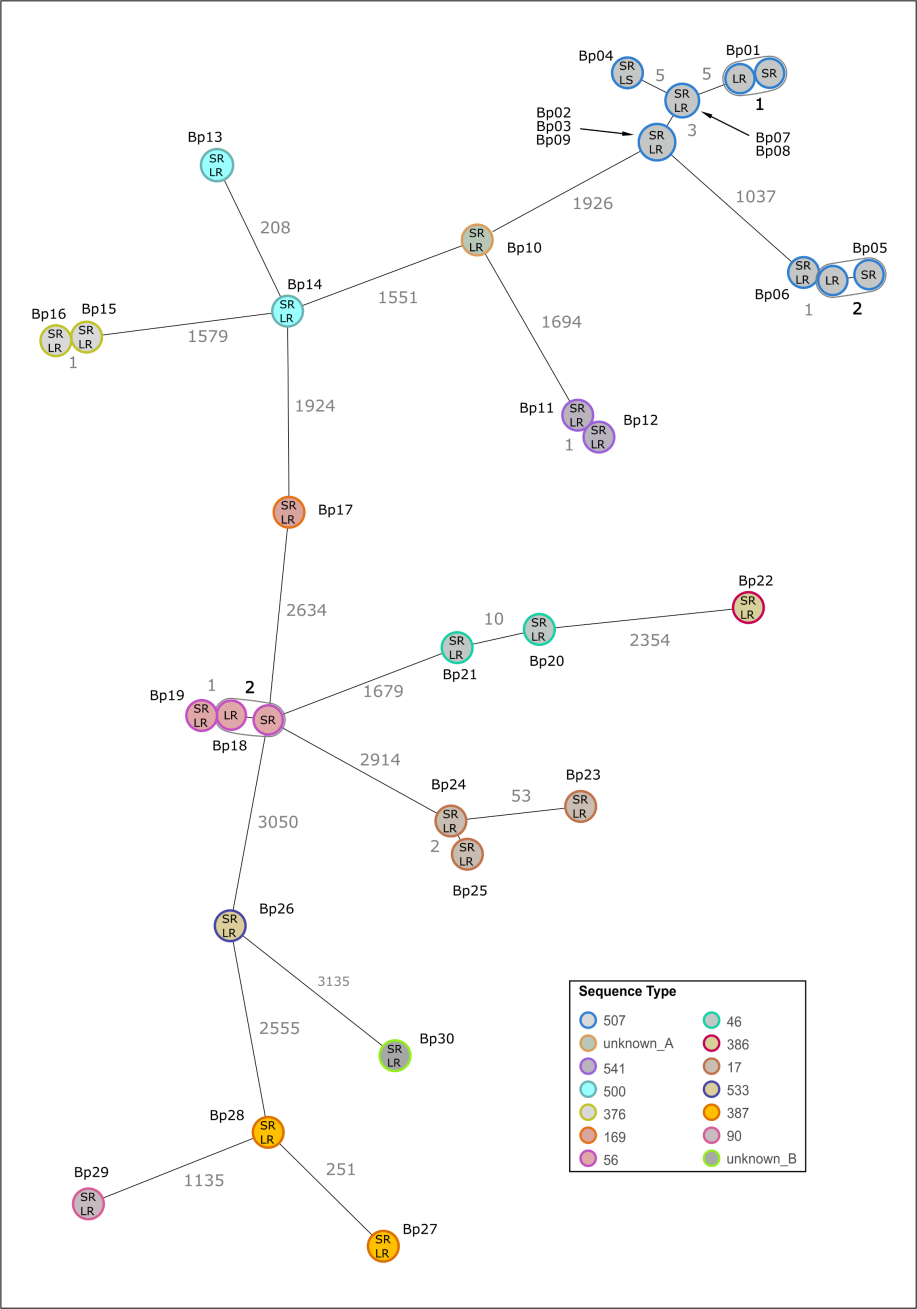
Comparison of ONT-LR cgMLST to the SR reference cgMLST. Thirty *B. pseudomallei* strains (Bp01-Bp30) were analyzed using ONT-LR (SUP@5.0, medaka_g360_HAC) and Illumina-SR based cgMLST. Each circle represents an allelic profile based on allele calling of 4,221 target genes. The numbers on the connecting lines refer to the number of allele differences. Allele differences between different strains are shown in gray, whereas allele differences between SR and LR data of one particular isolate are shown in black. Colors represent different STs. When ONT-LR cgMLST does not match the Illumina-SR reference, nodes are circled. Bp, *B. pseudomallei*; LR, ONT-LR cgMLST; SR, Illumina SR cgMLST.

Although SUP@v5.0 basecalling improved the error rate for all strains, it remained too high for reliable typing. Therefore, we investigated different polishing models to further minimize discrepancies in cgMLST typing between ONT-LR and Illumina-SR data. Polishing was performed on flye assemblies based on SUP@v5.0 basecalling since we identified it as the best performing model in the preceding step. Besides the recommended consensus medaka model for the respective basecalling model, we included its variant model and the model medaka_g360_HAC. Allele differences increased for medaka_consensus (average: 3.6; range: 0–60) when compared with unpolished data (average: 2.6; range: 0–33). In contrast, applying medaka_variant or medaka_g360_HAC decreased allele differences (medaka_variant: average: 0.4; range: 0–3 and medaka_g360_HAC: average: 0.2; range: 0–2) (Table S5). Racon polishing did not improve the typing error rate for medaka_g360_HAC model and increased the error for 6/30 strains using medaka_variant (Table S6). Of note, applying the same models to data generated after PCR-based library preparation had limited effects (Table S7). In conclusion, the highest number of typing errors was observed for the recommended polishing model (medaka_consensus), performing worse than unpolished assemblies. Medaka_g360_HAC outperformed all models and reduced the error rate to a maximum of two allele differences (Fig. 5 and 6). To further evaluate ONT-LR data, we conducted a core-genome SNP analysis based on the cgMLST targets for all 30 LR-assemblies and compared the results with the SR references and the cgMLST analysis (Fig. S1). SNP analysis revealed an identical number of differences (a maximum of two) between LR and SR data compared with cgMLST analysis, further supporting the robustness of LR data for bacterial typing.

### Rapid barcoding using the adapted pipeline

**Fig 7:**
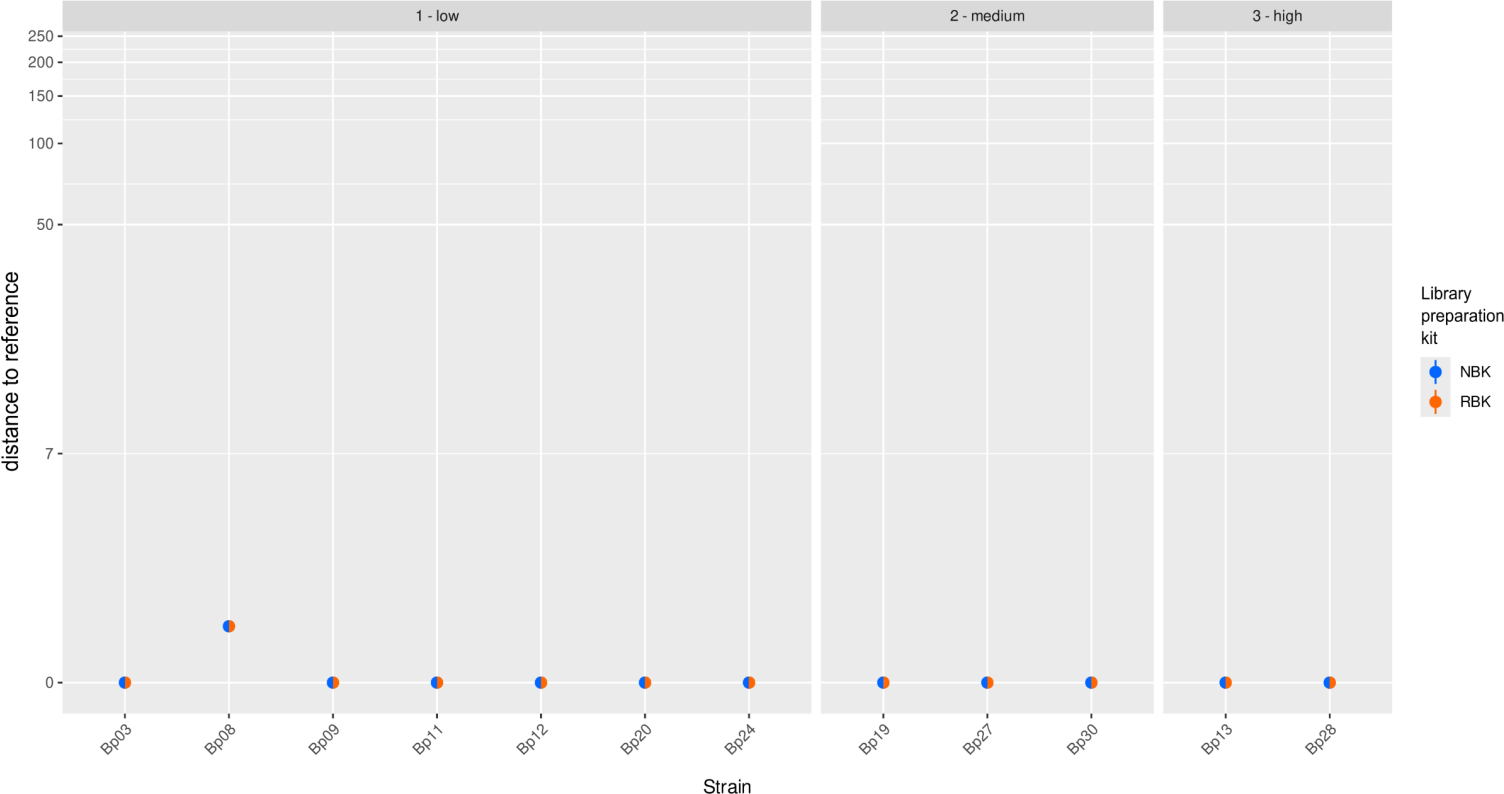
Allelic differences of the NBK- and RBK-based ONT-cgMLST in comparison to the SR reference. Dots in blue show the allele differences of the NBK (SUP@5.0 with medaka_g360_HAC)-based ONT-cgMLST compared with the SR reference. Dots in orange show the allele difference of the RBK (SUP@5.0 with medaka_g360_HAC)-based ONT-cgMLST compared with the SR reference. Mixed dots display overlapping results for the two ONT approaches. Bp, *B. pseudomallei*; NBK, native barcoding kit; PCR, PCR library preparation kit.

We next applied the adapted pipeline to rapid barcoding, representing an alternative for time-sensitive sequencing. Two of the “high typing error” (Bp13 and Bp28), three “medium typing error” (Bp30, Bp19, and Bp27), and six of the “low typing error” strains (Bp11, Bp03, Bp20, Bp24, Bp08, and Bp09) were re-sequenced. Applying the best performing pipeline identified for native barcoding (SUP@v5.0 with medaka_g360_HAC model) confirmed the improved results (Fig. 7; Table S8).

## DISCUSSION

Key demands for genomic pathogen surveillance include high accuracy, standardization, simple data analysis, and cost-efficiency. In theory, ONT-based cgMLST meets these criteria. However, the observation that some species display DNA methylation-induced typing errors (22) demands validation and optimization across species. PCR-based library preparation is suggested to resolve methylation-based errors (22). However, it is plausible that this approach might pose challenges to GC-rich bacteria. We addressed this issue by validating PCR-based library preparation using *B. pseudomallei* since it possesses one of the most complex bacterial genomes with 68% GC content. In addition to being a model for GC-rich bacteria, its role in causing a severe disease in many LMICs highlights the need for highly accurate, cost-effective typing. The initially performed ONT-cgMLST, relying on data generated using NBK and the at the beginning of this study, recommended pipeline (SUP@v4.2, medaka_consensus polishing), identified *B. pseudomallei* as challenging species for ONT-cgMLST. This is evident from almost 200 typing errors compared with gold standard Illumina-SR technology for some strains. Therefore, the implemented approach did not meet the demands for highly accurate typing since an error rate in this range will complicate source tracking and outbreak management. In line with other species (19, 22), our study indicates strain-specific typing difficulties for native barcoding. Importantly, strains within certain STs showed higher error rates compared to other STs. This underscores the fundamentality of validating ONT-based cgMLST across several genotypes of one distinct species and suggests methylation-based errors (22). This notion is strengthened by the fact that the application of a PCR-based library preparation resolved this issue to a certain degree.

To identify the most accurate ONT-cgMLST-based typing method, we validated PCR-based and native library preparation with modified data processing pipelines. Importantly, we included SUP@v5.0 while to the best of our knowledge, studies on other pathogens were limited to SUP@v4.3 or earlier versions. This is particularly important since SUP@v5.0 outperformed SUP@v4.3 in our study.

The implemented PCR protocol did not amplify the *B. pseudomallei* genome and needed adaptation for GC-rich DNA. The adapted PCR protocol led to a fragmented genome, demonstrating potential difficulties in reconstructing closed chromosomes and plasmids. Furthermore, the GC content did not match the expected one resulting from a PCR bias. Additionally, we observed an increase in missing targets. Although the PCR approach decreased the typing error rate for previously “high error rate” strains, it increased the error rate for previously unproblematic strains. In combination with the mentioned obstacles, the remaining 21 observed typing errors for some strains demonstrate that in the current state, PCR-based library preparation might not meet the demands for highly accurate strain typing of challenging GC-rich bacteria.

In parallel, we optimized data processing pipelines for NBK and RBK, which offer several advantages over PCR-based library preparation. These include decreased contig numbers, increased cost-efficiency, and reduced hands-on time. All adapted protocols led to a decrease in the initially observed error rate and outperformed PCR-based library preparation. The pipeline integrating SUP@v5.0 basecalling with medaka_g360_HAC polishing performed superior over all other tested pipelines, resulting in a maximum of two allele differences compared with the SR reference. In line with other reports (29), our data indicate that ONT-cgMLST does not benefit from racon polishing. Overall, this tremendous improvement in performance identifies ONT-based cgMLST as a valid alternative to SR-based cgMLST.

The fact that the adapted pipeline performed superior across all 27 tested genotypes underscores its general applicability for *B. pseudomallei*. Although we maximized strain diversity in available strains, future investigations could benefit from expanding the analysis to include additional strains as well as additional species. This would ensure further validation and reinforce the generalizability of our findings across an even broader context.

### Conclusions

PCR-based library preparation poses challenges to the tested GC-rich species and does not meet the demand for accurate strain typing as shown by our detailed investigations on *B. pseudomallei*. In contrast, native barcoding with an adapted data processing pipeline holds great promise reducing the typing error to a maximum of two allele differences. In conclusion, we provide a protocol enabling highly accurate as well as cost and time-efficient typing of *B. pseudomallei*. Furthermore, we describe a strategy allowing future investigations of other bacterial pathogens to improve global molecular surveillance by using a technology that is likely to be more widely accessible compared to SR sequencing.

## Data Availability

Sequencing data were deposited in the National Center for Biotechnology Information Sequence Read Archive repository under BioProject number PRJNA1161571.

## SUPPLEMENTAL MATERIAL

Supplemental material jcm.01569-24-s0001.pdf Tables S1 to S8; Figure S1.

ASM does not own the copyrights to Supplemental Material that may be linked to, or accessed through, an article. The authors have granted ASM a non-exclusive, world-wide license to publish the Supplemental Material files. Please contact the corresponding author directly for reuse.
