# Supplementary material for: Improving Nanopore sequencing-based core genome MLST for global infection control: a strategy for GC-rich pathogens like Burkholderia pseudomallei
Source: J Clin Microbiol. 2025 Feb 6;63(3):e01569-24. doi: 10.1128/jcm.01569-24 (PMC11898673; doi:10.1128/jcm.01569-24)
Supplement: Supplemental material — Tables S1 to S8; Figure S1. [file jcm.01569-24-s0001.pdf]

Online Supplement to:

Improving Nanopore sequencing-based cgMLST for global infection control: A strategy for GC-rich pathogens like *Burkholderia pseudomallei*.

Sarah Weigl, Johanna Dabernig-Heinz, Fabian Granitz, Michaela Lipp, Laura Ostermann, Dag Harmsen, Thanh Trung Trinh, Ivo Steinmetz, Gabriel E. Wagner, Sabine Lichtenegger

Table S1: Sequence type and cgMLST allele differences of ONT-LR genome assemblies based on the NBK and SUP@v4.2 basecalling with medaka_consensus polishing. ONT-LR data were compared with the SR reference. Strains were subdivided into three different typing error categories low (<5 allele differences), medium (5-100 allele differences) and high (>100 allele differences).

Strain	*Number of allele mismatches*	*Typing error category*	*Sequence Type*
Bp01	2	low	507
Bp02	0	low	507
Bp03	0	low	507
Bp04	0	low	507
Bp05	2	low	507
Bp06	0	low	507
Bp07	1	low	507
Bp08	1	low	507
Bp09	0	low	507
Bp10	0	low	unknown_A*
Bp11	4	low	541
Bp12	3	low	541
Bp13	160	high	500
Bp14	187	high	500
Bp15	1	low	376
Bp16	1	low	376
Bp17	0	low	169
Bp18	11	medium	56
Bp19	8	medium	56
Bp20	1	low	46
Bp21	0	low	46
Bp22	2	low	386
Bp23	1	low	17
Bp24	1	low	17
Bp25	3	low	17
Bp26	7	medium	533
Bp27	10	medium	387
Bp28	175	high	387
Bp29	1	low	90
Bp30	17	medium	unknown_B*
average	**20.0**		

*allele combination of ST unknown_A: *ace*-3, *gltB*-2, *gmhD*-2, *lepA*-3, *lipA*-5, *narK*-4, *ndh*-6; allele combination of ST unknown_B: *ace*-1, *gltB*-12, *gmhD*-6, *lepA*-2, *lipA*-1, *narK*-6, *ndh*-1

Table S2: Median GC content of reads generated after PCR-based library preparation and NBK. All reads were basecalled with the SUP@v4.2 model and analyzed with FastQC (v0.12.1).

Strain	***PCR, no alterations***	***PCR, Q5 polymerase + Q5 GC enhancer***	***NBK***
Bp13		65 %	68 %
Bp14		64 %	68 %
Bp16		66 %	67 %
Bp19		64 %	67 %
Bp20		65 %	67 %
Bp23	58 %	64 %	67 %
Bp25	58 %	65 %	67 %
Bp27		64 %	67 %
Bp28		64 %	67 %
Bp30		64 %	67 %
average	**58 %**	**64.5 %**	**67.2 %**

Table S3: cgMLST allele differences of ONT-LR genome assemblies based on PCR-based library preparation and SUP@v4.2 basecalling with medaka_consensus polishing. ONT-LR data were compared with the SR reference.

Strain	***Number of allele mismatches***
Bp13	21*
Bp14	14*
Bp16	1
Bp19	8*
Bp20	8
Bp23	12*
Bp25	6*
Bp27	10*
Bp28	15*
Bp30	14*
average	**10.9**

*missing values in more than 10% of target genes.

Table S4: cgMLST allele differences of ONT-LR genome assemblies based on NBK and different basecalling strategies without polishing. ONT-LR data were compared with the SR reference.

Strain	***SUP@v4.2***	***SUP@bacterial-methylation***	***SUP@v4.3***	***SUP@v5.0***
Bp01	2	1	1	1
Bp02	0	0	0	0
Bp03	1	0	0	0
Bp04	0	0	0	0
Bp05	2	2	2	2
Bp06	0	0	0	0
Bp07	1	1	0	0
Bp08	1	4	1	1

Bp09	1	0	0	0
Bp10	1	0	0	0
Bp11	6	2	0	0
Bp12	14	0	0	0
Bp13	278	66	29	14
Bp14	282	75	29	18
Bp15	1	0	0	0
Bp16	2	0	0	0
Bp17	0	0	0	0
Bp18	15	5	4	5
Bp19	16	4	1	2
Bp20	1	0	0	0
Bp21	1	0	0	0
Bp22	0	0	0	0
Bp23	1	0	0	0
Bp24	2	1	1	0
Bp25	3	1	1	0
Bp26	11	0	4	0
Bp27	11	3	7	2
Bp28	124	63	36	33
Bp29	2	0	0	0
Bp30	24	4	2	0
average	**26.8**	**7.7**	**3.9**	**2.6**

Table S5: cgMLST allele differences of ONT-LR genome assemblies based on NBK, SUP@5.0 basecalling with different polishing strategies. ONT-LR data were compared with the SR reference.

Strain	*No medaka*	*Medaka_consensus*	*Medaka_variant*	*Medaka_g360_HAC*
Bp01	1	1	1	1
Bp02	0	0	0	0
Bp03	0	0	0	0
Bp04	0	0	0	0
Bp05	2	2	2	2
Bp06	0	0	0	0
Bp07	0	0	0	0
Bp08	1	1	1	1
Bp09	0	0	0	0
Bp10	0	1	0	0
Bp11	0	3	0	0
Bp12	0	1	0	0
Bp13	14	5	1	0
Bp14	18	5	0	0
Bp15	0	1	0	0
Bp16	0	1	0	0
Bp17	0	0	0	0
Bp18	5	5	2	2
Bp19	2	0	0	0
Bp20	0	0	0	0

Bp21	0	0	0	0
Bp22	0	1	1	0
Bp23	0	0	0	0
Bp24	0	1	0	0
Bp25	0	0	0	0
Bp26	0	0	0	0
Bp27	2	20	0	0
Bp28	33	60	3	0
Bp29	0	0	0	0
Bp30	0	0	0	0
average	**2.6**	**3.6**	**0.4**	**0.2**

Table S6: cgMLST allele differences of ONT-LR genome assemblies based on NBK, SUP@5.0 basecalling, different medaka strategies and prior racon polishing. ONT-LR data were compared with the SR reference.

Strain	***Racon + Medaka_consensus***	***Racon + medaka_variant***	***Racon + medaka_g360_HAC***
Bp01	1	1	1
Bp02	0	0	0
Bp03	0	0	0
Bp04	0	0	0
Bp05	2	2	2
Bp06	0	0	0
Bp07	0	1	0
Bp08	1	1	1
Bp09	0	0	0
Bp10	1	0	0
Bp11	3	1	0
Bp12	3	0	0
Bp13	3	0	0
Bp14	3	1	0
Bp15	1	0	0
Bp16	1	0	0
Bp17	0	0	0
Bp18	5	2	2
Bp19	0	0	0
Bp20	0	0	0
Bp21	0	0	0
Bp22	1	2	0
Bp23	0	0	0
Bp24	2	0	0
Bp25	0	0	0
Bp26	0	0	0
Bp27	21	0	0
Bp28	65	3	0
Bp29	0	1	0
Bp30	0	1	0

average	**3.8**	**0.5**	**0.2**

Table S7: cgMLST allele differences of ONT-LR genome assemblies based on PCR-based library preparation, SUP@5.0 basecalling with different polishing strategies. ONT-LR data were compared with the SR reference.

Strain	***No medaka***	***Medaka_consensus***	***Medaka_variant***	***Medaka_g360_HAC***
Bp13	14*	19*	18*	20*
Bp14	20*	19*	21*	17*
Bp16	1	1	1	1
Bp19	5*	13*	9*	11*
Bp20	4	6	5	8
Bp23	2*	16*	3*	5*
Bp25	3*	9*	7*	5*
Bp27	7*	17*	12*	11*
Bp28	11*	14*	14*	21*
Bp30	5*	8*	8*	6*
average	**7.2**	**12.2**	**9.8**	**10.5**

*missing values in more than 10% of target genes.

Table S8: cgMLST allele differences of ONT-LR genome assemblies based on RBK, SUP@5.0 basecalling with different polishing strategies. ONT-LR data were compared with the SR reference.

Strain	No medaka	Medaka_consensus	Medaka_variant	Medaka_g360_HAC
Bp03	0	0	0	0
Bp08	1	1	1	1
Bp09	0	0	0	0
Bp11	0	0	1	0
Bp12	1	2	1	0
Bp13	10	1	5	0
Bp19	1	0	0	0
Bp20	0	0	0	0
Bp24	0	0	1	0
Bp27	3	12	2	0
Bp28	17	36	13	0
Bp30	1	3	0	0
average	**2.8**	**4.6**	**2.0**	**0.1**

cgMLST

cgMLST-SNP

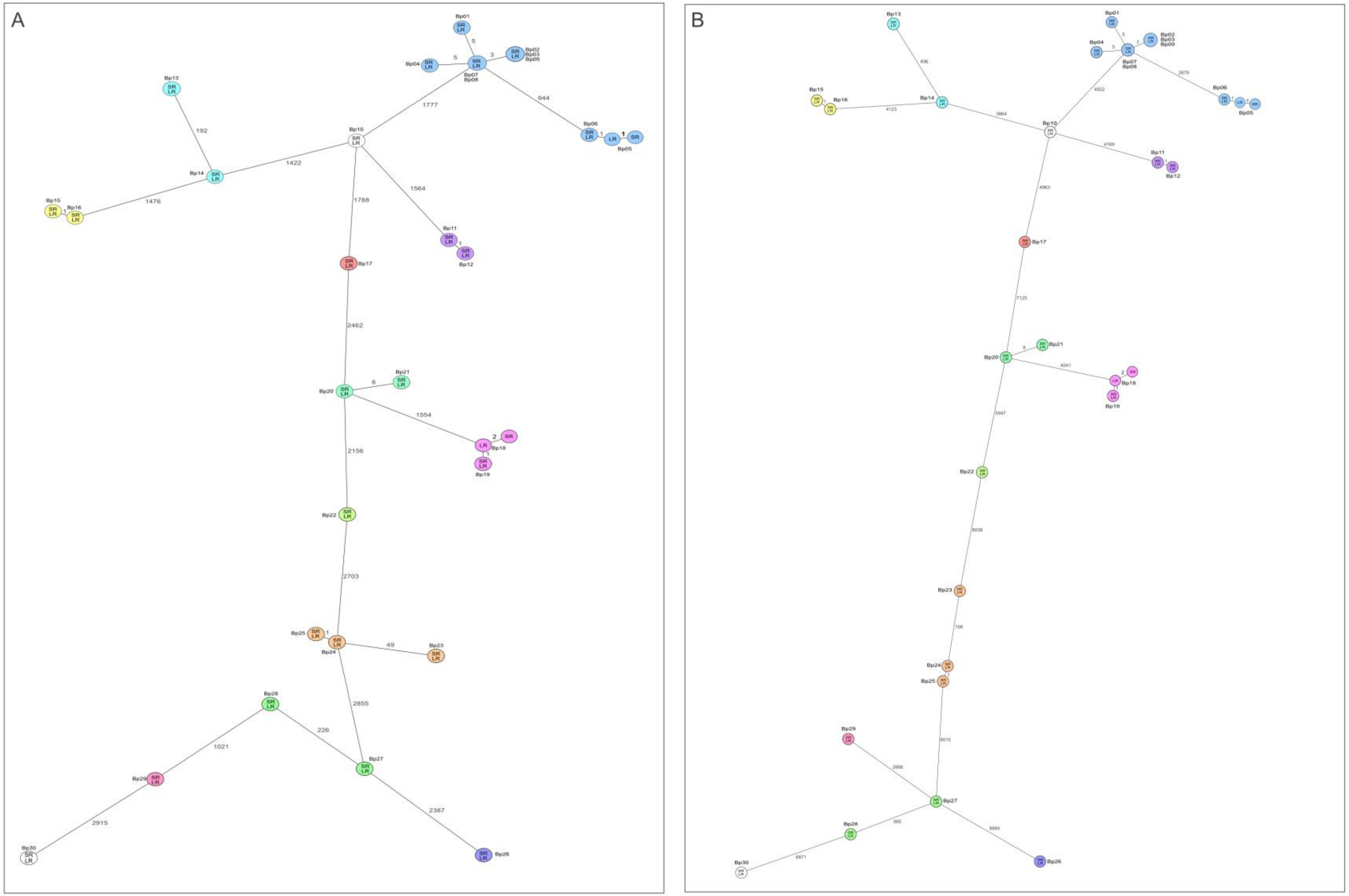

Supplementary Figure 1: Comparison of ONT-LR cgMLST and ONT-LR core genome SNP analysis including ONT-LR data as well as the SR reference. 30 *B. pseudomallei* strains (Bp01-30) were analyzed using ONT-LR (SUP@5.0, medaka_g360_HAC) and Illumina-SR based cgMLST (A). All 30 strains were additionally analyzed using ONT-LR core genome SNP analysis and Illumina-SR based core genome SNP analysis (B). Each circle represents a profile based on 3879 target genes. The numbers on the connecting lines refer to the number of allele/SNP differences. Allele/SNP differences between different strains are shown in gray, while allele/SNP differences between SR and LR data of one particular isolate are shown in black. Colors represent different STs. Bp, *B. pseudomallei*; LR, ONT-LR assembly; SR, Illumina SR assembly.
